# Population pharmacokinetics of artesunate and dihydroartemisinin following single- and multiple-dosing of oral artesunate in healthy subjects

**DOI:** 10.1186/1475-2875-8-304

**Published:** 2009-12-18

**Authors:** Beesan Tan, Himanshu Naik, In-Jin Jang, Kyung-Sang Yu, Lee E Kirsch, Chang-Sik Shin, J Carl Craft, Lawrence Fleckenstein

**Affiliations:** 1 College of Pharmacy, University of Iowa, Iowa City, IA, USA; 2 Department of Pharmacology and Clinical Pharmacology and Therapeutics, Seoul National University College of Medicine and Hospital, Seoul, Republic of Korea; 3 Shin Poong Pharmaceuticals Co., Ltd., Seoul, Republic of Korea; 4 Medicines for Malaria Venture, Geneva, Switzerland

## Abstract

**Background:**

The population pharmacokinetics of artesunate (AS) and its active metabolite dihydroartemisinin (DHA) were studied in healthy subjects receiving single- or multiple-dosing of AS orally either in combination with pyronaridine (PYR) or as a monotherapy with or without food.

**Methods:**

Data from 118 concentration-time profiles arising from 91 healthy Korean subjects were pooled from four Phase I clinical studies. Subjects received 2-5 mg/kg of single- and multiple-dosing of oral AS either in combination with PYR or as a monotherapy with or without food. Plasma AS and DHA were measured simultaneously using a validated liquid chromatography- mass spectrometric method with a lower limit of quantification of 1 ng/mL for both AS and DHA. Nonlinear mixed-effect modelling was used to obtain the pharmacokinetic and variability (inter-individual and residual variability) parameter estimates.

**Results:**

A novel parent-metabolite pharmacokinetic model consisting of a dosing compartment, a central compartment for AS, a central compartment and a peripheral compartment for DHA was developed. AS and DHA data were modelled simultaneously assuming stoichiometric conversion to DHA. AS was rapidly absorbed with a population estimate of absorption rate constant (Ka) of 3.85 h^-1^. The population estimates of apparent clearance (CL/F) and volume of distribution (V2/F) for AS were 1190 L/h with 36.2% inter-individual variability (IIV) and 1210 L with 57.4% IIV, respectively. For DHA, the population estimates of apparent clearance (CLM/F) and central volume of distribution (V3/F) were 93.7 L/h with 28% IIV and 97.1 L with 30% IIV, respectively. The population estimates of apparent inter-compartmental clearance (Q/F) and peripheral volume of distribution (V4/F) for DHA were 5.74 L/h and 18.5 L, respectively. Intake of high-fat and high-caloric meal prior to the drug administration resulted in 84% reduction in Ka. Body weight impacted CLM/F, such that a unit change in weight resulted in 1.9-unit change in CLM/F in the same direction.

**Conclusions:**

A novel simultaneous parent-metabolite pharmacokinetic model with good predictive power was developed to study the population pharmacokinetics of AS and DHA in healthy subjects following single- and multiple-dosing of AS with or without the presence of food. Food intake and weight were significant covariates for Ka and CLM/F, respectively.

## Background

Malaria is one of the deadliest infectious diseases in the world, causing nearly a million deaths among more than three billion people who were at risk in 2006 [1]. Unfortunately, given the high burden of the disease, the number of available anti-malarial drugs is relatively small. On top of that, the emergence of resistance to the most affordable anti-malarial drugs has seriously undermined the global effort to control malaria. As the result of chloroquine and sulphadoxine-pyrimethamine resistance, millions of lives that could otherwise be saved were sacrificed over the past 30 years [2]. Artemisinin-based combination therapy (ACT) is now being widely used as the first-line treatment for *Plasmodium falciparum *malaria throughout the world.

Artemisinin (ARN) and its derivatives, dihydroartemisinin (DHA), artesunate (AS), artemether and arteether are fast acting anti-malarial drugs producing the most rapid reduction in parasitaemia [3]. These agents also have gametocytocidal activity, which contributes to the reduction in the disease transmission [4,5]. Among the available derivatives, AS has the most appealing physicochemical and pharmacological properties. It is more water soluble, thermally and chemically more stable, and rapidly converted *in vivo *to its active metabolite DHA, which is responsible for most of the anti-malarial activity [6,7].

Pyronaridine (PYR) is a Mannich-base derivative anti-malarial that has been shown to be efficacious against erythrocytic stages of *P. falciparum *using *in vitro *models [8-10]. Clinical studies have also indicated that PYR is safe and efficacious against *P. falciparum *even in area with chloroquine-resistant strains [11-13]. Pyronaridine tetraphosphate plus artesunate (PA) is under development as a 3:1 fixed ratio combination for the treatment against *P. falciparum *and *P. vivax *malaria.

Population pharmacokinetics of AS and/or DHA following the administration of AS have been previously described in malaria patients. Karunajeewa and colleagues investigated the disposition of AS and DHA in 47 children from Papua New Guinea with uncomplicated malaria after the administration of AS suppositories [14]. The population pharmacokinetics of DHA were also assessed in 164 patients with moderately severe falciparum malaria following intra-rectal dosing of AS, in 24 pregnant women and in 70 African children with acute uncomplicated falciparum malaria after the administration of oral AS [15-17]. However, parent AS data were not modelled in these two studies, mainly because there were too few data points available for AS.

To date, no population pharmacokinetics analysis of AS and DHA has been published in healthy subjects receiving oral AS dosing. Such analysis will provide a means of comparing the pharmacokinetics of AS and DHA in malaria patients and healthy subjects and, therefore, expand the understanding in the effect of the disease state on the pharmacokinetics of AS and DHA. The aims of this analysis are to develop a population pharmacokinetic model of AS and DHA in healthy subjects following oral administration of PA combination or AS alone and to identify covariates that are important determinants of the variability seen in pharmacokinetic parameters of AS and DHA.

## Methods

### Subjects and study designs

Data used in performing this analysis were pooled from a four-part Phase I clinical trial (Protocol number SP-C-001-03). The trial was conducted at the Clinical Trial Center of Seoul National University, Seoul, South Korea, in accordance with the Guidelines of Good Clinical Practice and Declaration of Helsinki. Ethical approval was obtained from Institutional Review Board of Seoul National University. Written informed consent was obtained from each subject prior to the studies. All subjects were healthy Korean men and women evaluated by a physician at screening (Day -21 to -2) based on physical examination, vital signs, electrocardiogram, medical history and laboratory evaluations. All study drugs and placebos were provided by Shin Poong Pharmaceuticals (Seoul, Korea) and were identical in appearance to maintain blinding.

The first part was designed as a single oral ascending dose, randomized, double-blind, placebo-controlled, staggered and parallel group study to evaluate the pharmacokinetics, safety and tolerability of PA following single oral administration of PA at following doses: 6+2 mg/kg, 9+3 mg/kg, 12+4 mg/kg or 15+5 mg/kg. Nine subjects were recruited for each dose level, and randomized to receiving either PA treatment or placebo in 7:2 ratio.

The second part was conducted to evaluate the potential of drug interaction between PYR and AS when used as a combination in 3:1 ratio. It was a two-cohort parallel, two-period randomized, blinded, crossover study of a single oral dose of PA 12+4 mg/kg versus each of the individual drug at the same dose plus placebo of the other drug. 20 subjects were enrolled into this study, and randomly assigned into either cohort 1 or cohort 2 in equal numbers. In cohort 1, five subjects received PYR alone in period 1 followed by PA combination in period 2, while another five received the treatments in reverse order. In cohort 2, five subjects received AS alone in period 1 followed by PA combination in period 2, while another five received the treatments in reverse order. There was a 21-day washout period between the two periods.

The third study was a two-period, randomized and cross-over study to investigate the effect of food on the pharmacokinetics of PA. 20 subjects were enrolled into the study and randomly allocated to each of the fasted or the fed arm in equal numbers. After an overnight fast of at least 10 hours, the subjects in the fasted arm received PA 12+4 mg/kg with 240 mL of water and remained fasted for at least 4 hours post-dose while the subjects in the fed arm ingested a high-fat and high-caloric test meal 30 minutes prior to the treatment. After a washout period of 21 days, the subjects returned for the crossover treatment. Water was permitted as desired, except for one hour before and after drug administration, and a standard meal was scheduled at the same time in each period of the study for each subject.

The purpose of the last part of trial was to evaluate the pharmacokinetics, safety and tolerability of PA following multiple oral dosing of PA at following doses: 6+2 mg/kg, 9+3 mg/kg, 12+4 mg/kg or 15+5 mg/kg once daily for three consecutive days. The study design was otherwise similar to the design of the first study. Eight subjects were recruited for each dose level, and randomized to receiving either PA treatment or placebo in 6:2 ratio.

### Sample collection and storage

For the first three studies, venous blood samples for the determination of AS and DHA pharmacokinetics were collected prior to dosing and at 0.33, 0.67, 1.33, 1.67, 2, 2.5, 3, 4, 5, 8 and 12 hours post-dose. For the multiple-dosing study, the samples were taken prior to the dosing of each dose and at 0.33, 0.67, 1, 1.33, 1.67, 2, 2.5, 3, 4, 5, 8 and 12 hours after the third dose. Blood samples were collected into pre-chilled sampling tubes containing potassium oxalate/sodium fluoride (BD Vacutainer systems, Franklin Lakes, NJ) and placed on wet ice before centrifugation within five minutes of collection. Immediately after centrifugation, plasma was removed and transferred into two approximately equal volume aliquots in screw cap Nalgene cryovials and then frozen immediately at or below -80°C until analysis.

### Analytical method

Plasma concentration of AS and DHA were determined using a validated liquid chromatography-mass spectrometric method described by Naik *et al *[18]. All samples were assayed in the same laboratory. The plasma samples spiked with internal standard ARN was cleaned up using solid phase extraction method. Analysis was performed with a Shimadzu Model 2010 liquid chromatograph-mass spectrometer (Shimadzu, Columbia, MD) in single ion monitoring positive mode using atmospheric pressure chemical ionization as an interface. The lower limit of quantification for AS and DHA using 0.5 mL of plasma was 1 ng/mL. The coefficient of variation for intra-day and inter-day precision ranged from 7% to 14% and 9% to 14% for AS, and 11% to 14.9% and 11% to 15% for DHA, respectively.

### Population pharmacokinetic analysis

Nonlinear mixed-effect model building was conducted using NONMEM software version VI, level 2.0 (ICON Development Solutions, Ellicott City, MD) [19], as implemented on a Windows XP operating system (Microsoft Corporation, WA, Seattle) with G95 Fortran compiler (Free Software Foundation, Boston, MA). All models were fitted using the first-order conditional estimation method. NONMEM output was processed using PDx-Pop 3.10 (ICON Development Solutions, Ellicott City, MD) and Xpose version 4.0 (Uppsala University, Uppsala, Sweden) [20]. Graphical plots were produced using S-PLUS version 8.0 (Insightful Inc, Seattle, WA) and R 2.8.1 (Free Software Foundation, Boston, MA).

Measurements below the lower limit of quantification of the assay were excluded from the dataset. Since the molecular weights of AS and DHA are quite different (384.4 for AS and 284.9 for DHA), the concentrations were converted to the equivalent values in nmols/L. AS dose was also converted to the equivalent values in nmols. The concentrations were then natural log-transformed before the analysis.

Model selection was guided by the plausibility of the estimates, minimum objective function value (OFV), equal to minus twice the log-likelihood function, Akaike Information Criterion (AIC), equal to OFV plus two times the number of parameters, condition number, defined as the ratio of the largest Eigen value to the smallest Eigen value, visual inspection of diagnostic plots and the precision of parameter estimates.

In the initial stage of model building, one- and two-compartment pharmacokinetic models with first order absorption and first order elimination were fitted to the AS data to determine the best structural model for AS. Once the best pharmacokinetic model for AS was determined, DHA data was modelled as a metabolite compartment connected to the central compartment of AS. It was assumed that conversion to DHA was the only significant route of elimination for AS and that the conversion was irreversible [21]. One- and two-compartment models with first-order disposition were tested for DHA to develop the best metabolite structural model. For the two-compartment model, it was assumed that DHA was eliminated only from the central compartment. After the best structural model was determined, all parameters were estimated simultaneously using ADVAN 6 in NONMEM.

Inter-individual variability (IIV) of the pharmacokinetic parameters was modelled assuming a log-normal distribution, as follows:

where P_i _is the estimated parameter value for individual i, P_pop _represents the typical population estimate for the parameter and η_i _is the deviation of P_i _from P_pop_. The η random effects were assumed to be independent and symmetrically distributed with zero mean and variance ε^2^. A diagonal covariance matrix was modelled, as the data did not support the implementation of a full variance-covariance matrix. The magnitude of IIV was expressed as coefficient of variation (%CV), which was approximated by the square root of the variance estimate.

Residual variability (RV) was modeled using an additive model as shown below:

where C_ij _and C_pred, ij _represent the jth observed and model predicted AS or DHA concentrations, respectively, for individual i and ε_ij _denotes the additive residual random error for individual i and observation j. The ε random effects were assumed to be independent and symmetrically distributed with zero mean and variance σ^2^.

After the optimum model for AS and DHA was determined, covariate analysis was carried out to assess additional variables as possible determinants of the variability seen in the pharmacokinetic estimates. Covariates examined include total body weight, age, gender, type of dosing (single- or multiple-dosing), treatment (co-administration of PYR) and food intake (fasted or fed). A summary of the covariates evaluated is shown in Table 1. Potential covariates were initially identified using generalized additive modelling (GAM) as implemented in the Xpose software. The potential covariates were then tested using stepwise forward addition followed by stepwise backward elimination procedure [22]. The influences of the covariates were tested by adding a covariate to the model at a time in the forward addition step, and then by removing a covariate from the model at a time in the backward elimination step. The changes in OFV between the 'full' and the 'reduced' models were then calculated. The difference in OFV between two nested models was approximated by a χ^2 ^distribution. An OFV change of 3.84 (corresponding to a significance level of 5% at one degree of freedom) was used as the cut-off to include a covariate in stepwise addition. When no more covariates could be included, the stepwise backward elimination was carried out. For a covariate to remain in the model, a change in OFV of at least 10.83 (corresponding to a significance level of 0.1% at one degree of freedom) was needed.

**Table 1:** A summary of the study data, subject demographics and covariates included in the analysis.

Characteristic	Single dose study	Drug interaction study	Food effect study	Multiple dose study	Combined
No. of subjects	28	19	20	24	91
No. of concentration-time profiles	28	28	38	24	118
No. of observations					
AS	206	207	324	179	916
DHA	316	314	449	273	1352
AS dose (mg/kg)	2, 3, 4 or 5	4	4	2, 3, 4 or 5	2, 3, 4 or 5
Sampling schedule	Predose, 0.33, 0.67, 1, 1.33, 1.67, 2, 2.5, 3, 4, 5, 8 and 12 h postdose	Predose, 0.33, 0.67, 1, 1.33, 1.67, 2, 2.5, 3, 4, 5, 8 and 12 h postdose	Predose, 0.33, 0.67, 1, 1.33, 1.67, 2, 2.5, 3, 4, 5, 8 and 12 h postdose	Immediately prior to each dose and 0.33, 0.67, 1, 1.33, 1.67, 2, 2.5, 3, 4, 5, 8 and 12 h after the third dose	NA
Age (years)	24 (19-40)	23 (20-32)	21.5 (19-27)	23 (19-29)	23 (19-40)
Weight (kg)	62.5 (50.4-70)	60.9 (50.1-67.1)	59.9 (50.8-68.5)	62.2 (51.2-68.8)	61.5 (50.1-70)
Sex (number)					
Female	9	10	10	9	38
Male	19	9	10	15	53
Type of dosing	Single	Single	Single	Multiple (once daily for 3 days)	NA
Food intake (number of profiles)					
Fasted	28	28	19	24	99
Fed	0	0	19	0	19

AS, artesunate; DHA, dihydroartemisinin.

Continuous variables are given as median (range).

The relationship between continuous covariates and pharmacokinetic parameters were evaluated using both linear and power functions, with the covariates centered or scaled at their median values:

where θ_1 _represents the parameter estimate P of an individual with a body weight of 61.5 kg and θ_2 _is a factor describing the correlation between body weight and the parameter. The influences of binary covariates on the parameter were modelled using a proportional relationship, as follow:

where θ_3 _represents the parameter value in subjects receiving the test drug without food, and θ_4 _is the fractional change in the parameter in subjects receiving the test drug with food. FOOD variable was coded as 0 for the fasted subjects and 1 for the subjects who received test drug with food.

### Model evaluation

The non-parametric bootstrap procedure was employed to evaluate the precision of the parameter estimates and the robustness of the final model. 500 bootstrap datasets were generated by repeated random sampling with replacement from the NONMEM input data file, and the final NONMEM model was fitted to the bootstrap datasets. Bootstrap parameter estimates, standard errors and 95% confidence intervals were obtained and compared with the parameter estimates from the original dataset.

Visual predictive check was also performed to evaluate the predictive ability of the model. 500 virtual observations at each sampling time point were simulated using the final model and its parameter estimates. The observed data were then plotted with the 5th, 50th and 95th percentiles of the simulated data that was above the limit of quatification. The percent of observations outside the 90% prediction interval was also calculated. The condition number of the final model was also calculated as a measure of the stability of the model.

## Results

### Demographic data

Data from 118 concentration-time profiles arising from 91 healthy Korean subjects were pooled from four clinical studies. The subjects received single- or multiple-dosing of 2-5 mg/kg AS orally either in combination with PYR or as a monotherapy with or without food. A total of nine and 18 subjects contributed two pharmacokinetic profiles each in separate occasions for the drug-interaction and food effect study, respectively. Since the elimination half-lives for AS and DHA are very short and the elimination of the drugs are deemed to have completed after the 21-day washout period, the pharmacokinetic profiles arising from the same subject in different occasions were treated as independent profiles. 916 and 1,352 concentration measurements for AS and DHA respectively were used in the modelling. A summary of the study designs, subject demographics and covariates is shown in Table 1.

### Population pharmacokinetic model

A one-compartment model with first order absorption and first order elimination best described the AS data. When a two-compartment model was fitted to the AS data, minimum OFV and AIC were reduced moderately (11.897 and 3.897 unit, respectively). However, the visual inspection of goodness-of-fit plots showed no improvement in the fit. The precision of the estimates obtained were also slightly worse. Consequently, the simpler one-compartment model was used to fit the AS data.

The DHA data were then sequentially modelled using a one-compartment model with linear elimination and also a two-compartment model. The two-compartment model fitted the DHA data better, leading to165.186 and 157.186 unit reduction in OFV and AIC respectively. Goodness-of-fit plots also showed obvious improvement in the overall fit.

**Figure 1:**
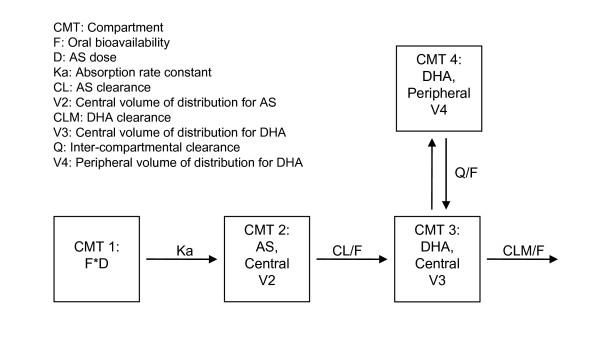
**Schematic representation of the final structural model**.

The final model used to simultaneously model the AS and DHA data consisted of a dosing compartment, a central compartment for AS, a central compartment and a peripheral compartment for DHA, as shown in Figure 1. The model was parameterized in terms of absorption rate constant for AS (Ka), apparent clearance for AS (CL/F, where F is the unknown oral bioavailability), apparent volume of distribution of the central compartment for AS (V2/F), apparent clearance for DHA from the central compartment (CLM/F), apparent central volume of distribution for DHA (V3/F), inter-compartmental clearance for DHA (Q/F), and apparent peripheral volume of distribution for DHA (V4/F). Inter-individual variability (IIV) was estimated for all parameters but Q/F and V4/F, since the available data would not support the inclusion of the two terms. Fixing the variance of the random effects for Q/F and V4/F to zero had little influence on the OFV, and was essential for the model to minimize successfully and to calculate the covariance matrix of the estimates. The population estimates of apparent clearance (CL/F) and volume of distribution (V2/F) for AS were 1190 L/h with 36.2% inter-individual variability (IIV) and 1210 L with 57.4% IIV, respectively. For DHA, the population estimates of apparent clearance (CLM/F) and central volume of distribution (V3/F) were 93.7 L/h with 28% IIV and 97.1 L with 30% IIV, respectively. The population estimates of apparent inter-compartmental clearance (Q/F) and peripheral volume of distribution (V4/F) for DHA were 5.74 L/h and 18.5 L, respectively.

Food intake (FOOD) and total body weight (WT) were found to be significant covariates on Ka and CLM/F, respectively, in the following relationships:

The typical value of Ka for subjects taken the test drug without food is 3.85 h^-1^. When the drug was taken with high fat and high caloric meal, Ka of AS was reduced by 84%. The inclusion of food intake as a covariate in the final model reduced the IIV of Ka from 135% to 112%, indicating that this covariate accounted for 31% of the variability on Ka. The apparent clearance of DHA, CLM/F was correlated with total body weight, in which a unit change in the weight would result in 1.9 unit change in CLM/F in the same direction. With the incorporation of weight in the final model, the IIV of CLM/F was reduced from 31.4% to 28%, thus accounting for 20% of the variability.

**Figure 2:**
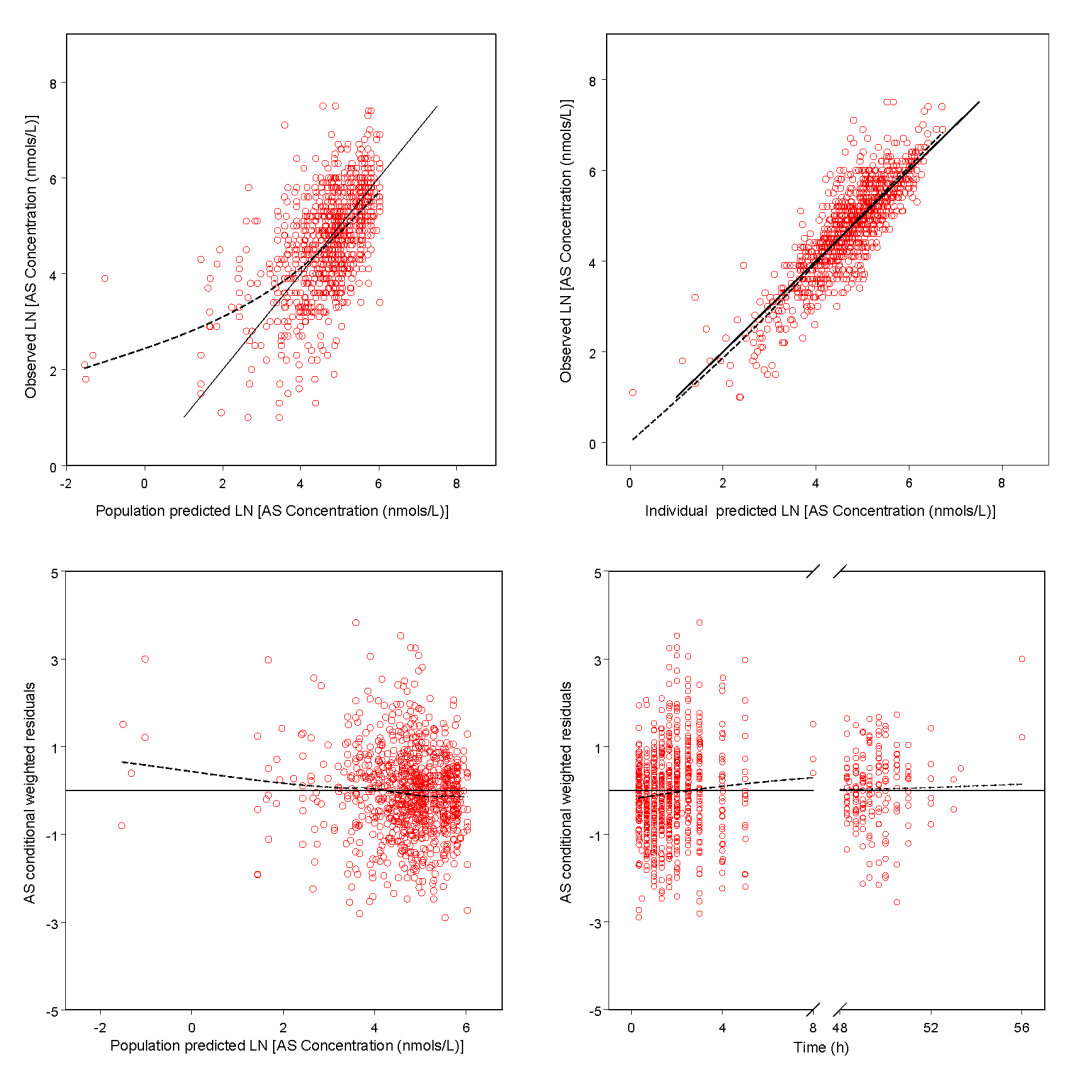
**Goodness-of-fit plots of artesunate (AS) for the final model**. The solid lines in the upper left and right panels are lines of identity. The broken lines are smoothing lines.

**Figure 3:**
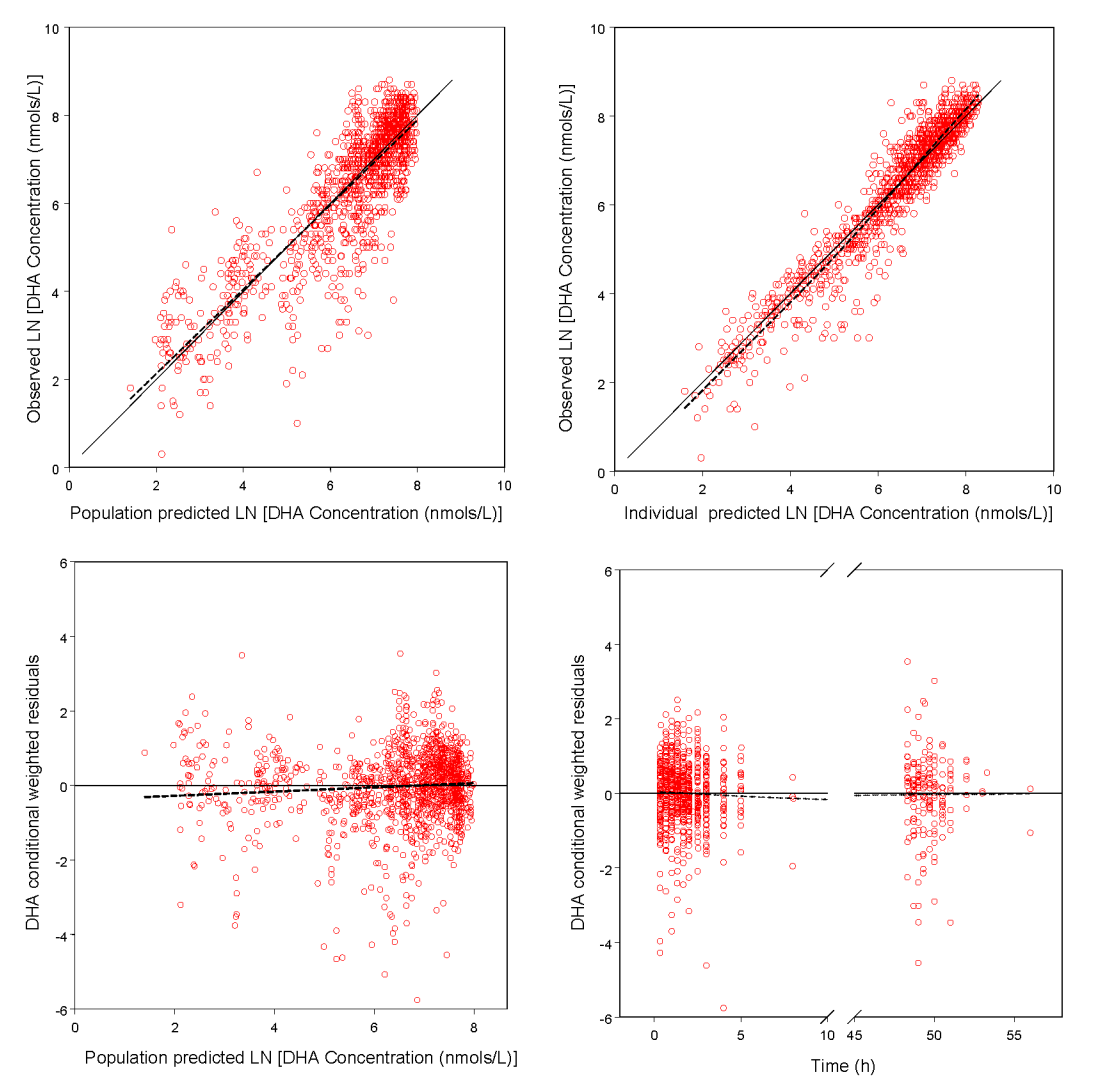
**Goodness-of-fit plots of dihyroartemisinin (DHA) for the final model**. The solid lines in the upper left and right panels are lines of identity. The broken lines are smoothing lines.

Goodness-of-fit plots for AS and DHA indicated a reasonable fit of the model to the data (Figure 2 and Figure 3). Final estimates of the parameters are shown in Table 2. The parameters were well estimated in general, with percent relative standard error ranged from 2.3% to 36%. Parameter related to absorption showed the most inter-individual variability, with IIV for Ka was estimated to be 112% even after the incorporation of food intake as a covariate. Residual variability was higher for AS observations than DHA observations.

**Table 2:** A summary of the results obtained from the final model and the bootstrap analysis.

Parameter	Estimate	%RSE	Bootstrap estimate (95% CI)
CL/F (L/h)	1190	4.20	1176 (1060 -- 1280)
V2/F (L)	1210	5.77	1199 (1020 -- 1370)
Ka (h^-1^)	3.85	3.61	4.16 (2.66 -- 6.67)
CLM/F (L/h)	93.7	3.30	92.6 (86.5 -- 99.1)
V3/F (L)	97.1	4.85	96.5 (86.7 -- 107)
Q/F (L/h)	5.74	12.8	5.69 (3.72 -- 7.53)
V4/F (L)	18.5	10.6	18.7 (14.1 -- 23.2)
θ_FOOD-Ka_	-0.84	2.32	-0.836 (-0.915 -- -0.733)
θ_WT-CLM/F_	1.90	16.3	1.78 (0.993 -- 2.58)
			
**IIV (Variances and % CV)**			
IIV-CL/F	0.131 (36.2)	17.8	0.130 (0.0824 -- 0.176)
IIV-V2/F	0.330 (57.4)	20.9	0.347 (0.201 -- 0.497)
IIV-Ka	1.26 (112)	15.4	1.32 (0.751 -- 2.00)
IIV-CLM/F	0.0786 (28.0)	22.5	0.0740 (0.0411 -- 0.118)
IIV-V3/F	0.0901 (30.0)	36.0	0.0776 (0.0001 -- 0.151)
			
**RV (%CV)**			
AS	37.5	9.73	37.5 (31.0 -- 45.2)
DHA	28.2	11.2	27.7 (21.6 -- 34.8)

RSE, relative standard error; CL/F, apparent clearance for AS; F, unknown bioavailability; V2/F, apparent volume of distribution of the central compartment for AS; Ka, absorption rate constant for AS, CLM/F, apparent clearance for DHA from the central compartment; V3/F, apparent central volume of distribution for DHA; Q/F, inter-compartmental clearance for DHA; V4/F, apparent peripheral volume of distribution for DHA; θ_FOOD-Ka_, parameter for the covariate food intake on Ka; θ_WT-CLM/F_, parameter for the covariate weight on CLM/F; CV, coefficient of variation; IIV, inter-individual variability; RV, residual variability; AS, artesunate; DHA, dihydroartemisinin.

### Model evaluation

**Figure 4:**
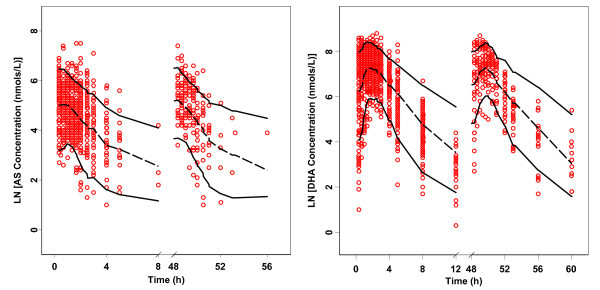
**Visual predictive check of the final model for artesunate (AS) and dihydroartemisinin (DHA) observations**. The open circles represent the observed concentrations, solid lines represent the 90% prediction interval obtained from the simulations, and the dashed line represents the 50th percentile of the simulations.

78% of the 500 non-parametric bootstrap runs converged successfully. The parameter estimates and 95% confidence interval for the parameters were calculated from the converged runs and are presented in Table 2. The parameter distributions were generally symmetrical. All the estimates obtained from the final model were comparable to the bootstrap estimates and were contained within the 95% bootstrap confidence intervals. Figure 4 shows the results of the visual predictive check for AS and DHA. Overall, the final model adequately described the observed concentrations. About 11.6% and 9.8% of the AS and DHA observations were not contained within the 90% prediction interval. The condition number of the final model was 24, indicating that the model was stable.

## Discussion

In the current analysis, a novel parent-metabolite model to describe the population pharmacokinetics of AS and DHA in healthy subjects was developed. This is the first population pharmacokinetic analysis of AS and DHA conducted using data derived from a large number of healthy subjects following oral administration of AS. The model developed was stable and was able to predict AS and DHA data arising from single- and multiple-dosing of oral AS equally well.

AS was rapidly absorbed into the systemic circulation, with an absorption half-life of 10.8 minutes estimated in this analysis. The conversion of AS to DHA was very rapid and the concentration of DHA was measurable as early as 20 minutes post-dose for all subjects. The sensitivity of the assay used in this study enabled the measurement of AS concentrations up to eight hours post-dose and the measurement of DHA concentrations up to 12 hours post-dose, in 65% and 95% out of the total 1416 available samples, respectively. Therefore, we were able to characterize the distribution of lipophilic DHA to the peripheral tissue. The pharmacokinetic parameter estimates obtained using a non-linear mixed-effects modelling approach employed in this analysis are comparable with those obtained using non-compartmental analysis (unpublished data).

The pharmacokinetics of AS and DHA following orally administered AS in healthy subjects have been previously reported in different settings [23-26]. However, AS pharmacokinetics was not described in the reports by Benakis *et al *[23] and Na-Bangchang *et a*l [26]. Navaratnam *et al *[24] compared the pharmacokinetics of AS and DHA after administration of oral and rectal AS in 12 healthy male Malaysian volunteers using non-compartmental approach. Following a single oral dose of 200 mg AS, the mean area under the plasma concentration-time curve (AUC) to time infinity for AS was reported to be 119 ng.h/mL, corresponding to a CL/F of 1680 L/h. A lower CL/F for AS (1190 L/h) was reported in the current analysis using data from a much larger sample size. The bioanalytical method used to measure AS plasma concentrations was also more sensitive (limit of quantification = 1 ng/mL) in current analysis. Teja-Isavadharm and colleagues [25] studied the single-dose pharmacokinetics of AS following the administration of 100 mg of oral AS in six healthy subjects and six patients with uncomplicated falciparum malaria. In their study, the range of apparent clearance and apparent volume of distribution in healthy subjects were 4.69-29 L/h/kg and 4.2-49.6 L/kg for AS, 1.66-3.26 L/h/kg and 1.99-4.45 L/kg for DHA, respectively. The weight normalized apparent clearance and apparent volume of distribution obtained in this analysis were 19.3 L/h/kg and 19.7 L/kg for AS, 1.52 L/h/kg and 1.88 L/kg for DHA, respectively. The estimates obtained in this present analysis are within the similar magnitude compared to their findings.

Several other studies have described the pharmacokinetics of DHA following the administration of oral AS in malarial patients [27-29]. Newton *et al *studied the disposition of DHA in 19 adult patients with acute uncomplicated falciparum malaria who were treated with 2 mg/kg of oral AS [29]. DHA data was modelled using an open one-compartmental model with first order absorption and elimination assuming complete conversion of AS to DHA. The reported CL/F and V/F values were 73.1 L/h and 70.5 L, respectively. In another study, the pharmacokinetic characteristics of DHA were described in 26 malarial patients who received 100 mg of oral AS, using non-compartmental analysis [28]. The reported AUC and t_1/2 _of DHA were 4.53 μmols.h/L and 0.66 h, respectively. These values correspond to a CL/F of 51.7 L/h and a V/F of 48.9 L. Similarly, Bethell *et al *reported the AUC and t_1/2 _of DHA of 1286 ng.h/mL and 1 h, respectively, in 10 children with moderately severe falciparum malaria who received 3 mg/kg of oral AS [27]. These values correspond to a CL/F of 45.7 L/h and a V/F of 65.9 L. All these values for CL/F and V/F of DHA were lower than the value reported in this analysis. These differences could be resulted from the use of different assays, the effect of disease state (malaria), differences in the dose formulations, and/or the age-related difference in DHA metabolism capacity.

Population pharmacokinetics of AS and/or DHA in malaria patients have been described in four other papers [14-17]. Karunajeewa *et al *[14] proposed a three-compartment model (a rectal absorption compartment, a central compartment for AS and a central compartment for DHA) to describe the population pharmacokinetics of AS and DHA simultaneously in paediatric patients following administration of AS suppositories. Simpson *et al *[16] modelled only the DHA data pooled from five Phase II and III studies conducted in adult and paediatric malaria patients. Both the weight-normalized CL/F and V/F for AS obtained in our analysis are much larger than the ones reported by Karunajeewa *et al *(5.9 L/h/kg and 2.1 L/kg, respectively) [14]. The larger CL/F and V/F for AS seen in our study might be attributed to the fact that the AS bioavailability is reduced when oral AS is given compared to rectal AS [24]. On the other hand, the weight-normalized CL/F and V/F for DHA obtained in this study are smaller than those reported by the two studies. Karunajeewa *et al *reported values of 2.2 L/h/kg and 2.1 L/kg for the CL/F and V/F of DHA [14]. The typical CL/F values of DHA reported by Simpson *et al *were 3.17 L/h/kg for a male and 2.03 L/h/kg for a female. For an adult weighted 70 kg, the typical value for V/F of DHA was 6.34 L/kg [16]. This finding is also consistent with the observations that the AUC for DHA following oral administration of AS was higher than that following rectal AS [24,30], suggesting that the bioavailability of DHA was increased when oral AS was given.

McGready *et al *[15] characterized the population pharmacokinetics of DHA in pregnant women with acute uncomplicated falciparum malaria following a three-day dosing of oral AS (4 mg/kg/day) and atovaquone plus proguanil. However, the pharmacokinetics of AS was not evaluated because AS was detectable only in about 6.5% of the total available samples. The pharmacokinetic parameter estimates for DHA were therefore derived using AS dose in DHA equivalents. The CL/F of DHA was reported to be 1.77 L/h/kg, which is similar in our analysis. However, the estimate for DHA apparent volume of distribution in healthy Korean subjects obtained in this analysis is about 60% lower compared to the pregnant Karen patients in their study (4.63 L/kg). The larger volume of distribution seen in their study might be attributed to the physiological changes during pregnancy and the effect of the disease state.

Stepniewska *et al *conducted a population pharmacokinetic study of AS in African children with acute malaria from six months to five years old [17]. The subjects received either the fixed dose combination of AS and amodiaquine or the separate tablets of both drugs. The DHA data was modelled using nonlinear mixed-effects approach. The weight normalized CL/F of DHA reported in their study was 0.636 L/h/kg, which was almost 60% lower than the value analysis in healthy adults. The discrepancy could be related to the developmental changes of metabolizing enzymes that take place in the young children. It has been demonstrated that the glucuronidation of DHA was catalyzed by UDP-glucuronosyltransferases (UGTs), in particular UGT1A9 and UGT2B7 [21]. The capacity of these metabolizing enzymes in young children could be much less than the full capacity in adults [31] and therefore resulted in lower CL/F of DHA. In a review on developmental patterns of UGT system, de Wildt *et al *suggested that the use of per-kg model for clearance is adequate to address developmental changes in young children and may lead the underestimation of clearance by up to 200% in chilren under 3.4 kg of body weight [31]. The reported weight-normalized V/F of DHA by Stepniewska *et al *was 2.285 L/kg [17], which was quite similar to the value obtained in this analysis.

In the present analysis, food intake was found to significantly delay the absorption of AS. When AS dose was administered after the intake of high-fat and high-caloric meal, the population absorption half-life of AS increased from 10.8 minutes to 67.5 minutes. However, the extent of absorption was not altered significantly. Body weight affected CLM/F significantly and therefore was included as a covariate in the final model. The average CLM/F for a healthy subject with 61.5 kg of body weight was estimated to be 93.8 L/h. A unit deviation in body weight would result in 1.9 unit deviation in the CLM/F from the population estimate. None of the other covariates tested was significant determinants of the variability seen. Co-administration of PYR did not affect any of the pharmacokinetic parameters of AS and DHA.

Remarkable time-dependent pharmacokinetics of ARN has been observed in both healthy subjects and in malaria patients after single or repeated oral and rectal administration of ARN dose [32-35]. Auto-induction of CYP2B6 and CYP2C19 was proposed to be the main mechanism causing the decline of plasma ARN concentrations [36-38]. A semi-physiological pharmacokinetic model taking into account the autoinduction phenomenon has been developed for ARN in healthy subjects [39]. Conflicting observations have been reported concerning the autoinduction phenomenon after the administration of AS dose. In their unconvincing report, Khanh *et al *[40] observed a decline in DHA concentrations in 6 malaria patients following repeated AS dosing. However, the decline in either AS or DHA concentration was not observed in two other studies [41,42]. In this analysis, the type of dosing was tested in the covariate analysis to investigate any differences in the pharmacokinetics of AS and DHA following the administration of single- and multiple-dose of AS. None of the AS and DHA pharmacokinetic parameters was affected by the type of AS dosing received at a significance level of 0.05. Visual predictive check plots in Figure 4 shows similar distributions for AS and DHA observations following single- or multiple-dose of AS, indicating no sign of decline in AS and DHA concentrations after three repeated daily AS dosing. The model described the AS and DHA observations equally well regardless the type of dosing received by the healthy subjects. In addition, the mean AS/DHA AUC_0-8 _ratios were similar after single and multiple doses (0.10 and 0.11, respectively). This provides evidence that there is little or no induction of the metabolic enzymes involved in the metabolism of AS following once daily dosing over three days.

## Conclusions

A descriptive, robust and predictive parent-metabolite model has been developed using population approach to characterize the pharmacokinetics of AS and DHA simultaneously in healthy subjects following oral administration of AS. In addition, presence of food and weight were found to impact the absorption and disposition of AS and DHA. The pharmacokinetic parameter estimates obtained in this analysis will also serve as a comparison for future works involving the characterization of population pharmacokinetics of AS and DHA following oral AS in malaria patients.

## List of abbreviations used

AS: Artesunate; DHA: dihydroartemisinin; PYR: Pyronaridine; ACT: Artemisinin-based Combination Therapy; ARN: Artemisinin; PA: Pyronaridine Tetraphosphate/Artesunate fixed combination in the ratio of 3:1; IIV: Inter-Individual Variability; RV: Residual Variability.

## Conflict of interests

J. Carl Craft was employed by the Medicines for Malaria Venture and C. S. Shin is employed by Shin Poong Pharmaceutical Co. Ltd as stated in the affiliations.

## Authors' contributions

BT conducted the pharmacokinetic modelling and drafted the manuscript. HN conducted the bioanalysis of the artesunate plasma samples. IJJ and KSY carried out the clinical aspects of the study including the acquisition of data. BT, HN, LF and LK participated critically in discussions throughout the modelling process and interpretation of pharmacokinetic data. IJJ, CSS, JCC and LF contributed substantially to the concept and design of the clinical trial and were part of the protocol development team. All authors critically reviewed the manuscript and approved the final version of the manuscript for submission.
